# Severe and enduring mental health problems within an established substance misuse treatment partnership

**DOI:** 10.1192/pb.bp.113.045138

**Published:** 2014-10

**Authors:** Kathryn Walsh, Alex Copello

**Affiliations:** 1 University of Birmingham; 2 Birmingham and Solihull Mental Health Foundation Trust

## Abstract

**Aims and method** The study reports findings of an investigation into the presence of severe and enduring mental health problems within the four statutory and non-statutory teams of an established substance misuse treatment partnership.

**Results** Of a total of 772 clients in the four teams surveyed, 69 (8.9%) were identified as having severe and enduring mental health problems and problem substance use in the past 12 months. Alcohol was the most prevalent substance used by this predominantly male group. Different rates were found across the four teams, with higher numbers in the non-statutory teams. The clients displayed significant levels of self-harm and suicide risk and were responsible for 131 acute service contacts over the past 12 months.

**Clinical implications** Clients with severe and enduring mental health problems engaged with substance misuse services display high levels of complex need. It is important to identify the best and most effective service response to this group.

‘Dual diagnosis’, the coexistence of mental illness and substance misuse, has attracted considerable amount of interest over recent years. Prevalence studies in the UK have primarily focused on substance misuse among clients within mental health services,^1-6^ with less consideration being given to the presence of mental health problems, particularly those which are severe, within substance misuse services. The figures that are available vary greatly, ranging from 4 to 85%;^7-10^ this could be a result of differences in the geographical area of the study,^10^ service type and the severity of mental health problems and substance misuse identified.

Higher rates of mental health problems are identified within substance misuse services when the inclusion criteria are broad; for example, 75% of drug and 85% of alcohol service clients were identified as having a mental health disorder in the past year.^9^ When focusing on severe and enduring problems, however, the number falls as low as 4%.^7^ The Comorbidity of Substance Misuse and Mental Illness Collaborative (COSMIC) study, with a higher estimate, reported non-substance-induced psychotic disorders as present in 8% of drug and 19% of alcohol service clients.^9^

The present study aimed to establish: (a) the presence and type of severe and enduring mental health problems, and (b) the substances used by this group, within the four component statutory and non-statutory teams of an established substance misuse treatment partnership serving a geographical population. In addition, presenting mental health problems and main misused substances were compared between the four service teams.

## Method

### Sample

Data were collected across an established substance misuse service partnership in an area of the UK serving a population of 95 000. The partnership comprised four services: a statutory National Health Service (NHS) community drug team, a non-statutory community alcohol service, a non-statutory community drug service and a statutory psychiatric liaison service based within a general hospital accident and emergency department. A method was developed from that used by Graham *et al*.^7^ Within each of the three community services all staff with an active case-load were invited to be involved in the survey and provide information on each client on their current case-load. A total of 28 staff were recruited of a possible 29 (96.6%). Due to the service configuration within the psychiatric liaison service and the absence of staff case-loads, one member of staff was recruited to establish the client group that presented at the service within the past month. The 14 staff surveyed within statutory services were a combination of nurses (*n* = 7), including community psychiatric nurses, drug workers (*n* = 6) and one social worker. Within non-statutory services all 14 members of staff were drug (*n* = 4) or alcohol (*n* = 10) practitioners. Practitioner staff were from mixed professional backgrounds and often had qualifications in counselling or social work in addition to specialist training in addiction-specific interventions. The complete data-set contained information on 772 of a possible 808 clients (95.5%) seen by the partnership.

### Data collection

Following approval of the study, all members of staff with an active case-load were approached to take part and met individually with a researcher to discuss their current case-load. During the meeting the researcher completed a copy of the adapted prevalence questionnaire for each client (described in detail in the next section) in a structured interview format. All questionnaires were pre-coded by the researcher to ensure client anonymity.

### Measures

A three-section prevalence questionnaire^7^ was minimally adapted and used for the present study. The first section collects demographic information about the client, for example gender, age and ethnicity. The second section is based on a modified version of the Clinician Rating Scales for Alcohol (CAUS) and Drug Use (CDUS).^11^ These scales are based on DSM-IV diagnostic criteria for substance-related disorders and are used to reliably classify the severity of substance use among individuals with severe mental health problems.^11^ Clinicians are asked to rate the pattern of substance use for the identified client across a range of substances for the past 12 months, on a scale of 0 (abstinent) to 6 (dependence with institutionalisation). In a previous study,^7^ two partial tests of the reliability of these ratings against standardised substance use measures showed levels of agreement to be moderate to good. For the purpose of this study, this section of the questionnaire was slightly modified to include the frequency of use for each substance (number of days per week) and the client’s primary substance of misuse. The third section of the measure records hospital admissions and home treatment episodes, self-harm and suicidal ideation, and reasons for substance use.

The first section of the questionnaire was completed for all clients. If the client had a severe and enduring mental health problem or had experienced psychotic symptoms, and misused substances in the past 12 months, the second and third sections of the questionnaire were also completed. For the purpose of the present study, severe and enduring mental health problems were defined as psychotic disorder, bipolar disorder and major mood disorder.^7^

## Results

### Demographic characteristics of the sample

The clients across the four services were predominantly male (*n* = 529, 68.5%) and had a mean age of 40 years (s.d. = 12.29, range 18-90). A younger case-load was captured within the non-statutory drug service (mean 31 years, s.d. = 7.61) and an older one within the non-statutory alcohol service (mean 46 years, s.d. = 11.50). The majority of clients (*n* = 709, 91.8%) across the services were considered to be White (British or Irish) and this was consistent across the four teams. The clients had been in substance misuse services for an average length of 2 years and 4 months (range 1 day to 15 years) and had been working with their current keyworker for an average length of 1 year (range 1 day to 10 years).

### Coexisting substance use and severe mental health problems

Of the 772 clients, 69 (8.9%) were identified by their keyworker as having a severe and enduring mental health problem and as having misused substances in the past 12 months. The presenting mental health problems included psychosis (*n* = 39, 56.6%), major depression (*n* = 22, 31.9%) and bipolar disorder (*n* = 8, 11.6%). This ‘dual diagnosis’ sample was demographically similar to the whole study sample, with a mean age of 40 years (s.d. = 9.54, range 21-63) and being more likely to be White (*n* = 65, 94.2%) and male (*n* = 42, 60.9%).

Of these 69 clients, 47 (68.1%) had a formal diagnosis of a mental illness and 22 (31.2%) did not. Some clients presented with multiple diagnoses, which may have also included coexisting anxiety disorders or personality disorders. Of the 47 clients with a formal diagnosis, more than half had a primary diagnosis of psychotic disorder (*n* = 26) and around a third major depressive disorder (*n* = 16). A smaller number had a primary diagnosis of bipolar disorder (*n* = 5). These diagnoses were documented within the clients’ clinical case notes and were provided by a psychiatrist. The clients without a formal diagnosis were usually in the process of being referred into or assessed by mental health services, and often presented with symptoms suggestive of a psychotic disorder (*n* = 13), a depressive disorder (*n* = 6) or bipolar disorder (*n* = 3). These presenting mental health problems were identified by the client’s keyworker using a set of standard criteria replicated from previous research.^7^ The clients without a formal diagnosis had been in the services on average 6 months less than those with a formal diagnosis.

Overall, alcohol was the most prevalent substance across the sample (*n* = 52, 75.4%), followed by cocaine (*n* = 30, 43.5%) and opiates (*n* = 20, 29.0%). A smaller number of clients were using cannabis (*n* = 15, 21.7%). The majority of clients were using substances to a problematic level (ratings of use with impairment, dependence or dependence with institutionalisation) and a high number of clients were using more than one substance problematically. With regard to primary substance used, alcohol was the most prevalent (*n* = 40, 58.0%), followed by opiates (*n* = 13, 18.8%). Table 1 shows the mental health and substance use problems by client group in both statutory and non-statutory services.

### Level of severe mental health problems and substance use within each substance misuse service

The level of severe mental health problems varied across the four services, with higher rates in the non-statutory teams (drug service: *n* = 16, 17.2%; alcohol service: *n* = 32, 14.0%) compared with the statutory teams (NHS drug team: *n* = 16, 4.4%; psychiatric liaison service: *n* = 5, 5.6%). Clients within statutory services were more likely to have a formal diagnosis of a mental illness (drug service: *n* = 13, 81.2%; psychiatric liaison service: *n* = 4, 80.0%) than those within non-statutory services (alcohol service: *n* = 19, 59.4%; drug service: *n* = 11, 68.8%).

**Table 1:** Primary substance use according to primary presenting mental health problem within both statutory and non-statutory substance use services (*n* = 69)

	*n* (%)					
	*n*	Alcohol	Cannabis	Opiates	Cocaine	Other
Statutory services^a^	21					
Psychotic disorder^b^	14	3 (21.4)	-	5 (35.7)	3 (21.4)	3 (21.4)
Major mood disorder^c^	3	3 (100.0)	-	-	-	-
Undiagnosed mental health problems	4	1 (25.0)	-	2 (50.0)	1 (25.0)	-
						
Non-statutory services^d^	48					
Psychotic disorder^b^	12	8 (66.7)	-	2 (16.7)	2 (16.6)	-
Major mood disorder^c^	18	12 (66.7)	2 (11.1)	3 (16.7)	1 (5.6)	-
Undiagnosed mental health problems	18	13 (72.2)	1 (5.6)	1 (5.6)	1 (5.6)	2 (11.2)

a. A National Health Service (NHS) community drug service and an NHS psychiatric liaison service within a general hospital accident and emergency department.

b. Includes schizophrenia, psychosis, schizoaffective disorder and delusional disorder.

c. Includes depressive disorders and bipolar affective disorder.

d. A non-statutory community alcohol service and a non-statutory community drug service.

The presenting mental health problem varied by team, with higher rates of psychosis in the statutory services (*n* = 16, 76.2%) than the non-statutory services (*n* = 23, 47.9%), and higher rates of depression in the non-statutory services (*n* = 19, 39.6%) compared with the statutory services (*n* = 3, 14.3%). Similar levels of bipolar disorder were observed across services.

With regard to substance use, problematic use of alcohol as a primary substance was higher within the non-statutory services (*n* = 33, 68.8%) than the statutory services (*n* = 7, 33.3%). The statutory services, however, had higher rates of opiate and cocaine use.

### Risk factors

Of the 69 clients with coexisting problems, 59 (85.5%) had contact with acute services (psychiatric hospital or home treatment teams) in the past 12 months. The number of acute service contacts ranged from 1 to 22 per client (mean 1.98, s.d. 4.07), with a total of 131 over a 12-month period. There was considerable variation in the total number of acute service contacts across the four substance misuse services, with the smallest number being from the NHS community drug team (*n* = 2) and the largest from the non-statutory alcohol service (*n* = 93). In total, the client group within non-statutory services had 115 contacts, compared with 16 from those in the statutory services.

Forty-nine (69.6%) clients were identified as having self-harmed or having suicidal ideation in the past 12 months. Again there was variation across the four teams, with the client group within non-statutory services more likely to have self-harmed or expressed suicidal ideation (*n* = 40, 83.3%) compared with those within the statutory services (*n* = 8, 38.1%).

## Discussion

The results of the present study can add to the existing literature given the focus on a whole substance misuse treatment system, including statutory and non-statutory teams, that provides an overview of the level of severe and enduring mental health problems across different service components within a defined geographical area. In terms of the overall number of clients with coexisting severe mental illness and substance misuse, the rate found in this study falls somewhere between prevalence estimates reported by Graham and colleagues^7^ and those found by Weaver *et al*.^9^ However, a more detailed exploration found different rates between statutory and non-statutory teams and these are discussed further below, after consideration of the issue of diagnosis and the substances used by those with severe mental health problems.

The issue of diagnosis of mental health problems in people who misuse alcohol or drugs is difficult, and often a presenting mental health problem may be directly related to the substance use, for example, a result of intoxication or withdrawal. This would therefore require an alternative care pathway and highlight a different service need for a client than would an enduring mental health problem that is part of a ‘dual diagnosis’. Through the identification within substance misuse services of clients who have a formal severe and enduring mental health diagnosis provided by a psychiatrist, this problem was somewhat diminished; however, for approximately a third of the sample no formal diagnosis was available. In these cases the client’s presenting mental health problem was identified by their keyworker who may not have been experienced or equipped to separate a substance-induced mental health problem from an enduring mental health problem. Within the study, attempts to minimise this over-identification risk included the use of set criteria to guide the keyworker’s assessment of the client.

When examining the range of substances used by those with severe mental health problems, partly in line with previous findings within this group, the most frequently reported substance was alcohol. The main substances used by these clients fell in line with the types of substance-focused services that each participating team provided. Overall, the one substance that appeared reported at lower rates was cannabis. The most likely reason for this could be that the services taking part focus mainly on alcohol and opiates with some response provided for stimulants and cocaine, but no robust treatment response is offered to those using cannabis as a primary drug. Given that we know cannabis to be one of the most prevalent substances used by those with combined severe mental illness and substance misuse,^2,7,12,13^ this finding may highlight a gap in service provision within our study area that may also be present in other UK services. The findings suggest that although individuals who use cannabis and have a diagnosis of serious mental health problem are more likely to be in contact and engaged with severe mental health problem services, those for whom a mental health problem has not been identified but who are using cannabis may not be engaged in substance misuse services and hence have no contact with services at all. This means that early identification of risk and service response to those using cannabis prior to the identification and diagnosis of the severe mental health problem may be more difficult in current service configurations.

As mentioned, different rates of severe mental illness and substance misuse problems were found between the statutory and non-statutory teams. Rates were lower in the statutory services (both community and liaison), whereas they appeared higher in both non-statutory teams. Schulte and colleagues^10^ reported that non-statutory substance misuse teams were more likely to accept clients with coexisting mental health problems than were statutory teams. However, it is also possible that the route into mental healthcare teams is easier to activate for those substance misuse teams who are already part of a statutory treatment system, and hence these teams may find it easier to place these clients within mental health services earlier. Based on the current findings, it is not possible to clarify the reason for this difference, but it is an important area for future research and service planning. In addition, the differences found between statutory and non-statutory teams highlight the need to consider the whole treatment system within an area, as any prevalence obtained from only one type of service (e.g. statutory) will only reflect a partial picture or estimate.

Integrated treatment within mainstream mental health services is recommended for clients with severe mental illness and coexisting substance use.^14^ At one level, it could be argued that all of the clients identified in this study should be placed within mental health services that deliver robust integrated treatment for both mental health and substance use and therefore should not be within the specialist substance misuse services. However, this creates a dilemma, as it is unlikely that robust integrated treatment is in place in all areas, and where it is not available or robust, substance misuse services provide at least some response to the substance-related problems experienced.

It is therefore possible that there are a number of clients within both statutory and non-statutory substance misuse services across the UK who are complex and costly to manage and who are not having the full range of needs met within the service where they are placed. It is important to identify the best service placement and response for this client group. It was evident from our findings that this group’s healthcare use through acute and in-patient services was high and therefore costly, and attempts to reduce these healthcare costs through a more integrated response would be worthwhile.
